# How can academic researchers more effectively contribute to environmental toxicology and health efforts for regulatory decisions, policymakers, nonprofits, and communities?

**DOI:** 10.1080/10937404.2026.2636513

**Published:** 2026-03-09

**Authors:** Joel N. Meyer, Elizabeth F. Boxer, Kevin M. Crofton, Richard T. Di Giulio, Allie Gartland-Gray, Amelia M. Foley, Laura E. Jameson, Chiara Klein, Nishad Jayasundara, Rashmi Joglekar, Reshma Nargund, Samantha M. Samon, Elizabeth Shapiro-Garza, Heather M. Stapleton, Paige M. Varner, Tenley A. Weil, Charlotte R. Clark

**Affiliations:** a Nicholas School of the Environment, Duke University, Durham, NC, USA; b Integrated Toxicology and Environmental Health Program, Duke University, Durham, NC, USA; c Center for Data Interoperability, RTI International, Research Triangle Park, NC, USA; d Program on Reproductive Health and the Environment, University of California, San Francisco, CA, USA; e Environmental Defense Fund, Raleigh, NC, USA

**Keywords:** Fundamental research, applied research, research translation, community engagement, systematic reviews

## Abstract

Most ecological and human environmental health researchers are motivated both by curiosity and a desire to do work that will be useful sooner rather than later. However, the academic research process does not always produce and present results that meet the needs of those we imagine using it beyond other researchers. The aim of this review was to discuss needs and opportunities associated with research question development, experimental design, data sharing, science communication, and community engagement, with the goal of supporting regulators, policymakers, environmental nonprofits, and pollution-impacted communities. For example, each regulatory agency has unique policies regarding study characteristics required before the agency includes the study results in its policy-making. To illustrate the extent of this problem: systematic reviews used by policymakers often keep only approximately 5% of papers originally found, because others fail to meet inclusion criteria of which academics are often unaware. The review also details the importance of data sharing via databases and science communication and opportunities for engagement with policy-makers, nonprofits, and communities, and obstacles that researchers face in conducting research that generates data useful to such groups. Our findings demonstrate that while not all academic research can or needs to be designed to be quickly applicable, opportunities exist where this *is* possible with relatively minor changes to typical academic practices. It is hoped that this review will help identify ways that academic researchers might both address fundamental, basic research knowledge gaps and contribute more directly and rapidly to policy making and community needs.

## Introduction and context: good environmental health science is critical for policy

Quantitative toxicity data, exposure measurements, and understanding of fate and transport of chemicals are typically used to support regulatory policies and, for some agencies, are mandated. For example, while such data are required to register chemicals through the Federal Insecticide, Fungicide, and Rodenticide Act (FIFRA), they are not required under the amended Toxic Substances Control Act (TSCA). Rather, the “best available science” needs to be evaluated in addition to the “weight of the scientific evidence” (US Environmental Protection Agency 2017). Importantly, rational reasons exist for the different requirements: FIFRA governs pesticides that are intentionally applied to crops, soil, and potentially food and are designed to be bioactive (i.e., control pests), whereas TSCA regulates industrial chemicals, many of which are used in controlled settings and/or with less direct consumer exposure. However, the current framework does not always provide the protection needed for vulnerable ecosystems and communities in a timely manner, in part because sufficient robust and quantitative data for many chemicals and mixtures are lacking. Approximately 40,000 chemicals are active in US commerce, with an estimated 350,000 chemicals and chemical mixtures in commercial production and use globally. For the majority of these, sufficient information and data to assess their potential hazards is lacking (Pelley 2020). It should be noted that the amount of information required may vary based on usage volume. Approximately 40% of chemicals are used in relatively low levels (1–10 metric tons), where physical-chemical properties, acute toxicity, and genotoxicity are assessed but other endpoints may not be required; for larger volume chemicals, greater information requirements exist (ECHA, n.d. (see Supplemental References)). Further, regulation of chemicals through statutes like the Toxic Substances Control Act (TSCA) might take several years, and agencies often regulate individual chemicals at a time. Since TSCA was amended in 2016, fewer than 10 chemicals have been banned or restricted in the US. Community groups, nonprofits, and others are often motivated to take action on their own, but likewise often lack relevant data on chemical pollution to take informed action.

Importantly, the challenge of many data-poor chemicals and mixtures is a global reality, but the approach to assessment and management varies from country to country. For example, in a Canadian context, regulatory frameworks for chemicals assessment require the use of weight of evidence but also precaution in the absence of information; similarly, a precautionary principle-based approach is more emphasized in Europe. In Canada, all available information must be considered, including vulnerable populations, vulnerable environments, and cumulative effects.

Academic researchers have an opportunity to look for ways to effectively fill these data gaps to both drive effective regulatory decisions and inform communities that may be directly impacted by the research. In addition to collection of quantitative data, this may include other practices and processes, such as use of qualitative data, culturally responsive and equitable evaluation methods as applied to the environmental health sciences, and community-based science (Askew, Beverly, and Jay 2012). Critically, academic contributions have the potential to inform and improve environmental health protection globally, given the international challenges of pollutant effects on health (Fuller et al. 2022) and the global need for toxicological information to inform risk assessment noted above.

Given the enormous number of chemicals and mixtures currently in use, academicians may wonder which to focus on. Academic researchers may wish to seek guidance from EPA and other agencies involved in chemical regulations on which data are most needed to inform sound policy. An example of how this can be helpful is evident in the recent academic science contributions to regulatory decision-making on per- and polyfluoroalkyl substances (PFAS). Scientific contributions to not just the toxicological effects of individual PFAS exposures but also to the effects of PFAS mixtures built the foundation for the final National Primary Drinking Water Regulation for six PFAS in 2024 (US Environmental Protection Agency, 2024 (see Supplemental References)). This was the first drinking water standard to utilize a hazard index approach to regulating mixtures of chemicals that might result in adverse health effects when combined in a mixture, even if the chemicals do not elicit effects individually at lower levels. It should be noted that food tolerances for pesticides with common modes of action have also been used for some time in cumulative risk decisions (US Environmental Protection Agency, Office of Pesticide Programs (2002, 2011). Further, and as also highlighted by the PFAS case, as the fields of toxicology and environmental health move toward cumulative risk assessments and chemical mixtures analyses that reflect real-world exposure scenarios, little consensus exists as to how data from these studies can impact decisions in current regulatory policy frameworks that evaluate health and environmental impacts on a chemical-by-chemical basis. As researchers adopt methodologies that characterize hazard and risk from exposure to chemical mixtures, there is an urgent need to ensure that experimental data is actionable and can support and strengthen chemical policy that better protects public health. Academic researchers have the opportunity to design their experiments in consultation with public health and policy experts (including scientific experts from environmental nonprofit organizations like Earthjustice and Environmental Defense Fund), and members of communities impacted by toxic chemicals who often have the best understanding of the multiple stressors that they experience and can help identify the critical knowledge gaps. While this review focuses in particular on the United States context given the authors’ experience, such opportunities exist elsewhere as well. For example, Canadian stakeholders including the both researchers and the general public can engage with policy-makers, nominate chemicals for consideration, and voluntarily submit information/data in the context of the Plan of Priorities of the Canadian Environmental Protection Act (Environment and Climate Change Canada, 2025a (see Supplemental References)).

The uncertainty and continual evolution of science can be difficult to fit into the often rigid and slow-changing policy sphere. Conversely, in some cases, regulatory decisions need to be made without sufficient scientific knowledge, as when COVID masking protocols were enacted on state and local levels relying largely on mechanistic reasoning, despite having no prior randomized trial data (Marasinghe 2020; Muller 2021). Similarly, proactive policies may be required in some cases to reduce harm from emerging contaminants with limited data.

The political incentive to delay regulatory action until the scientific research is complete and a scientific consensus has been reached may prolong exposures and deepen health inequities, particularly for residents of communities where chemical plants or waste sites have been sited nearby (i.e. fence-line communities). Fenceline community residents, often low-income and/or people of color, are more likely to bear the dual burden of higher chemical and non-chemical stressors, including poor access to healthcare and healthy foods, increasing their susceptibility to harm from chemical exposures (Gee and Payne-Sturges 2004; Johnston and Cushing 2020; Mohai and Saha 2015; Morello-Frosch et al. 2011; Smith et al. 2022).

In one example of chemical and nonchemical stressors, fenceline communities in and around Houston, Texas (Harris County) have some of the highest pollution rates in the country coupled with historical inequities that result in the life expectancy of these communities being 20 years lower than more wealthy and white neighborhoods within the same county (Abraha, 2019; Owens, 2019; (see supplemental references)). Zip codes and/or Census tracts with the lowest life expectancies in Harris County are highly burdened by chemical exposures that often exceed federal guidelines. Residents of these communities also experience risk factors associated with social determinants of health, such as poor access to quality healthcare, education, food, housing, and supportive social and community structures, and economic instability. For example, the Galena Park community in East Houston is in the 99th percentile of Census tracts across the country regarding vulnerability to industrial pollution sources (i.e., in the most-exposed 1%), coupled with being 99th and 91st percentile in the country for chronic disease prevention and access to healthcare, 88th percentile for socioeconomic stressors such as education, poverty, and violent crime, and 98th percentile for transportation sources such as. proximity to ports and rail crossings, and noise from transportation (Environmental Defense Fund, 2025(see supplemental references)). Another community that highlights these inequities is in Wilmington, North Carolina, where GenX and other PFAS were discovered in the municipal drinking water. This occurred because a chemical manufacturer upstream released wastewater containing PFAS into the Cape Fear River starting in the 1980s. As a result, some areas in this community, such as Census tract 37,129,010,800, rank in the 97^th^ percentile for pollution, and the health inequities are heightened by high rates of unemployment, low-income households, and violent crime (Tee Lewis et al. 2023)

**Figure 1.**
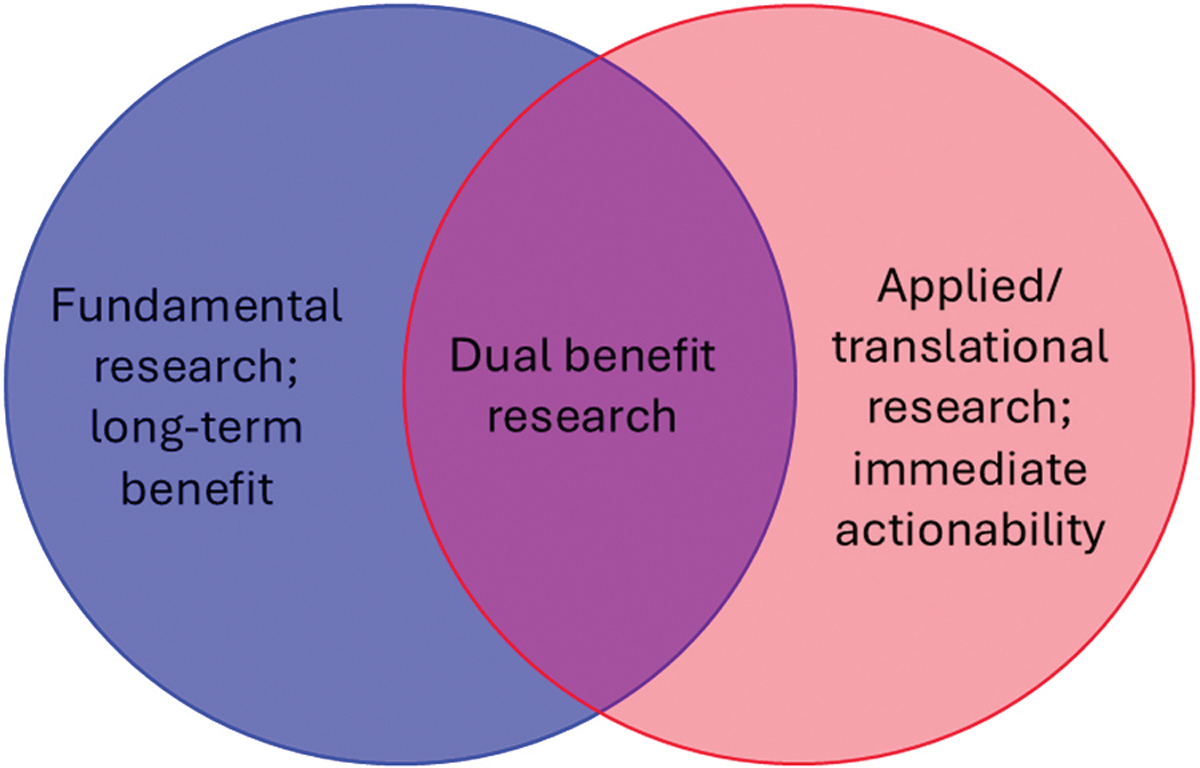
Dual benefit research produces both new fundamental knowledge with long-term benefits, and new data and knowledge that can be applied in the short term to immediate problems.

Given the importance of not only high quality scientific research, but also effective communication of said research to inform and drive policy decisions, a critical question is how researchers, in particular academic investigators, might better contribute. This review summarizes some insights regarding identification of key research questions and experimental design that fill policy gaps, increasing data availability, effectively translating and integrating research into policy, effective engagement with communities and regulators, and obstacles researchers may encounter. In some cases and with thoughtful planning and execution, researchers might indeed contribute to both the generation of fundamental knowledge with long-term benefits, the traditional goal of basic academic research, and generate data and knowledge that is immediately actionable (Figure 1). To be clear, “immediately actionable” is not mean to suggest that non-peer-reviewed data be used for action; but rather that peer-reviewed academic research might in some cases be conducted and communicated in a way that both addresses fundamental knowledge gaps (e.g., understanding the basic biology of DNA repair) and provides data that may be useful for more immediate regulatory purposes (e.g., characterizing the mutagenicity or lack thereof of new chemicals). A list of speakers, panelists, and moderators who contributed to these insights in a day-long Symposium held at Duke University in 2023 is provided in the acknowledgments; they are also named in this manuscript as appropriate.

## Identifying research questions

Academic training, particularly in the natural sciences, is often geared to identify questions whose answers address fundamental knowledge gaps, but not necessarily to identify questions that inform regulatory efforts or address societal problems. While enormous value exists in fundamental, basic research to push boundaries of knowledge, and these often have exceptional long-term benefits (Cairney and Oliver 2017; Fazey et al. 2013; Head 2022; Marshall 2025; National Academies of Sciences, Engineering and Medicine, 2017 (see supplemental references)), cases exist where researchers might create research questions that *also* address real-world issues and drive meaningful change in the shorter term. Increasing investigative focus on societally relevant research questions might help scientists to bridge the gap between scientific knowledge and practical application (Corburn 2007; Minkler, Vásquez, and Shepard 2006; Nadadur et al. 2007). To contribute more immediately to environmental health efforts, when possible, academic researchers need to identify questions whose answers are useful in the short term to regulators, policymakers, and community-based groups. This section highlights two ways to form research questions collaboratively: direct communication with public health and policy experts, decision-makers, and/or community groups, and the use of systematic reviews and preexisting databases to identify knowledge gaps

Scientists might build relationships and collaborations that inform the development of policy- and community-informed research questions. Dr. April Kluever, a senior toxicologist in the Office of Management and Budget (OMB), described the benefits of academic scientists developing research questions that result in actionable data to inform health policy, as well as opportunities to be involved in the processes of developing science-related mandates and risk assessments. Through direct communication and engagement with public health experts (e.g. scientific experts at environmental NGOs) and decision-makers (e.g. state or federal regulatory agency staff, elected officials), investigators might identify inflection points or gaps in science that may play a critical role in how decision-makers ultimately regulate chemicals. For instance, which chemicals and mixtures are most important? For a given chemical or mixture, what are the greatest uncertainties in the existing hazard or exposure information that might be addressed with new data? From the perspective of a regulatory or policy process, answering these questions might inform the prioritization of chemicals for hazard or risk assessment in the scoping phase in which regulators decide what chemicals to evaluate and what questions to ask (Pennell et al. 2013). Even in the absence of direct communication with regulators, academics may examine regulatory guidance documents, scientific strategies, and assessment framework documents, both nationally and internationally (e.g., the Organization for Economic Co-operation and Development 2025; OECD n.d.) to see where information is insufficient and/or where there is need for support in research areas such as emerging science, alternative methods, and validation.

Most chemical regulatory statutes require rulemakings to occur within a specific time frame, and the dataset available to support these rulemakings is rarely as complete as desirable. In these situations, a regulatory decision must be made anyway; frequently, agencies will acknowledge the uncertainty associated with incomplete data in its final regulatory decision. These uncertainties may be seen as data gaps that need to be addressed by new research. For example, after the passage of the Food Quality Protection Act, the Office of Pesticide Programs was required to consider the cumulative risk posed by mixtures of pesticides with a common mode of action (US Environmental Protection Agency 2002) At that time, there was significant uncertainty about the hazards of exposures to mixtures of pyrethroid insecticides. This uncertainty fostered an internal EPA Office of Research and Development (ORD) research program that developed extensive dose-response and toxicokinetics data, as well as human-relevant exposure modeling to predict the effects of pesticides mixtures, including pyrethroids (Hughes et al. 2016; Starr et al. 2012; US Environmental Protection Agency, ORD 2006; 2010, Wolansky et al. 2009). This is one of several research programs created to address uncertainties from data gaps (i.e., research needs) in regulatory agency decisions that was highly unlikely, at the time, to be an NIH-funded academic project.

To provide an academic example, in the last competitive renewal grant application submitted by the Duke University Superfund Research Center (DUSRC), consultations with the DUSRC External Advisory Board that includes EPA and community members illuminated both the importance of evaluating chemical mixtures and their effects on neurodevelopment, and identifying and generating data to address uncertainties in the body of evidence that might inform and strengthen chemical risk assessment. This knowledge guided development of the DUSRC’s research goals. Such approaches need not be entirely chemical-focused. For instance, investigators might also ask which aspects of Adverse Outcome Pathways (AOP), or biological pathways related to the development of chronic diseases (Ankley et al. 2010) used by regulators to evaluate health hazards, are the least certain. Investigators might then carry out basic mechanistic research to support, refute, or refine those uncertain portions of the AOP (Leist et al. 2017; Markovich et al. 2022; Morton, George, and Meyer 2025, Tebby et al. 2022; Teeguarden et al. 2016). In one example, the explicit statement in an AOP (Tebby et al. 2022) that *in vivo* evidence was lacking motivated experiments to test the AOP *in vivo* (Huayta and Meyer 2025). Researchers might also derive research questions from final regulatory decisions, as the documents describing these decisions (e.g. TSCA chemical risk evaluations or risk management rules) often point out the uncertainties in supporting scientific assessments. These uncertainties, which may include lack of adequate exposure measurements or lack of information on the toxicokinetics or toxicodynamics of a chemical, may be used to drive applied research projects that might readily impact risk decisions.

Similarly, through communication with community groups, scientists might develop research questions that may directly address societal and human health concerns. The DUSRC Community Engagement Core (CEC) does this by engaging in bi-directional communication with communities and research partners, providing a pathway for these groups to bring their concerns and questions to scientists. Through such approaches, investigators may develop research questions that address the needs of the community in ways that lead to substantive change through education, outreach, and/or advocacy. For example, in 2016, an informal community group in the Lower Cape Fear Region of North Carolina noted individuals fishing from the Cape Fear River, a known impacted waterway (Chiara Klein and Elizabeth Shapiro-Garza, Duke SRC, pers. comm.) for which several fish consumption advisories exist (NC Department of Health and Human Services, Division of Public Health, 2026 (see Supplemental References)). This group became concerned regarding adverse health impacts from consuming the fish they were catching and recruited the CEC to help them apply for an EPA Problem Solving Grant to address the concern. In the years since, DUSRC investigators conducted research on the contaminants of concern, and the CEC has stayed actively involved in the efforts to educate the fishing community about chemical contaminant exposure through wild-caught fish. This involved the CEC working closely with a coalition of local groups to develop such resources as a social marketing campaign, informational surveys, a white paper, a social media toolkit, and educational videos, and to facilitate activities including community forums, webinars, additional fish tissue testing, and report-backs (Shapiro-Garza, 2025; Shapiro-Garza et al., 2022 (see Supplemental References)).

An important consideration is which academic specialists need to be on the team engaging with the community. Examples might include an exposure scientist (environmental or personal measurements), public health expert/epidemiologist (what outcomes are of concern?), toxicokinetics expert (understanding exposure, distribution, metabolism, and excretion), toxicologist (e.g., researcher evaluating Molecular Initiating Eventss, Key Events, or adverse outcomes in the AOP), etc. Participants may evolve over time.

Another way in which researchers can identify policy-relevant knowledge gaps is through the use of systematic reviews, which are structured reviews on the complete body of evidence on a certain topic (Rooney et al. 2014). Dr. Xabier Arzuaga (US EPA) described systematic reviews, which are an essential part of the risk assessment paradigm. The use of a validated, gold-standard systematic review method might help investigators understand the full body of evidence on a topic and identify research questions that address relevant knowledge gaps (UCSF Program on Reproductive Health and the Environment, 2025 (see Supplemental References)). Using Population of interest, Exposure, Comparator, and Outcome (PECO) criteria is a critical first step in formulating a scientific question that clarifies the objectives of a systematic review. By utilizing this process, researchers might form clear questions for a systematic review to address how specific environmental exposures affect health outcomes (Morgan et al. 2018). To illustrate, Dr. Julia Rager, an academic researcher, completed a systematic review that combined articles from peer-reviewed literature (academic sources) with data from the Environmental Protection Agency ToxCast database (Dix et al. 2007), to determine what exposures to chemicals of concern are currently unexplored (Carberry and Rager 2023). After such a review, locations of communities that might be exposed to these chemicals may be prioritized for research (including community-based participatory research), community education, and/or policy considerations.

By aligning their research with the needs and concerns of community members, public health experts and/or decision-makers, scientists may better balance the desire to produce information that contributes to basic scientific understandings, the traditional goal of academic research, with generating actionable data that informs evidence-based policy decisions, improves public health, and enhances community well-being.

## Incorporating policy and regulatory needs into experimental design and results reporting

Systematic Reviews are also used by regulatory scientists as a critical first step in chemical risk assessment, although different agencies may use somewhat different criteria to find, select, and judge research (National Academies of Sciences, Engineering, and Medicine 2021). However, these criteria often differ in part from those academics are trained to consider as “good” in the context of their research design and communication. While conducting high-quality research is of primary and overarching importance, this section highlights other aspects of how academic researchers might enhance the likelihood that results of such excellent research may be used in the process of regulation and policy development.

Academic researchers might often make their research more likely to be included in policy and regulation by making relatively small changes to experimental design and results reporting. This is critical because systematic reviews often retain only approximately 5% of the papers originally identified in the literature after applying inclusion criteria (Uttley et al. 2023). For example, a recent systematic review of diisobutyl phthalate initially found 1184 potentially relevant papers that were put into the review pipeline; however, by the end, only 19 (<2%) were considered of high enough quality and relevance to be included in the compiled analysis (Yost et al. 2019). The reasons for non-inclusion are not always under the control of researchers, but in many cases they are.

In some cases, these considerations are simply a matter of explicitly communicating specific methods ordinarily employed in the research. For example, random assignment of test samples (cells, animals, etc.) to experimental groups is a widely used and expected aspect of experimental design for all researchers. Blinded assessments for quantitative and qualitative analyses, such as scoring of microscopic images, are also routine, and software programs exist to facilitate blinded scoring (Cothren, Meyer, and Hartman 2018). However, these practices are so common among scientists that methods sections of research manuscripts often do not explicitly state that random assignation and blinding were used. Failure to state that they were used is frequently a reason for exclusion from consideration in risk assessment. Similarly, to avoid having their studies excluded from systematic review, researchers need to explicitly describe quality control measures such as exposure characterization (e.g., test material identification, purity analysis and characterization, and exposure measurements), verification of biological samples (e.g., verifying that cells are uncontaminated and are in fact the cell line described, typically with genetic testing); present all positive and negative controls; use multiple dose levels to examine dose-response; ensure adequate sample sizes; and provide control of confounding variables. Inclusion of animal welfare data (e.g., body weights, clinical signs) is important to determine whether the maximum tolerated dose was exceeded, as are cell viability/cytotoxicity assessments in *in vitro* studies. Finally, publicly filing protocols in advance of study work might add clarity to the status of the investigation. To avoid having their studies excluded from agency systematic reviews, researchers should, at minimum, explicitly include and describe these measures in their studies, especially since none are likely to change most experimental designs.

Other relatively small adjustments to experimental design and data reporting might increase the likelihood of one’s research being included in agency scientific assessments. This includes designing studies to include a range of exposure concentrations, at a minimum 3–5, and publishing the full range of data, including data that are often considered “range-finding” (Meyer and Francisco 2013). In general, all data needs to be reported in full, which is now required by the NIH in any case (National Institutes of Health, n.d.-a (see Supplemental References)). Similarly, methods need to be described fully and in detail; of course, this has long been a scientific expectation, but this has been hindered historically by page limits on publications. This is another case where good, reliable scientific practice – reporting methods in sufficient detail that other scientists can precisely replicate them – is in perfect alignment with making data more likely to be use used by regulators and policy-makers. Further, this is much easier now given the widespread and easy availability of databases and repositories for “supplemental” material, raw data, metadata, and methodological detail, whether hosted by the journal or elsewhere. Results for all methods used need to be reported, including any negative findings. Exposure levels (concentrations and doses – ideally including internal, measured concentrations that permit comparison to human exposure data) that are environmentally relevant should be included if feasible. When possible, following OECD or EPA methods guidelines in planning and conducting research increases the likelihood of meeting inclusion criteria. For example, for *in vitro* methods, guidance is provided by OECD No. 421 (OECD, 2025 (see Supplemental References)). Furthermore, several journals now explicitly invite negative data papers, for example: micropublications Biology and Royal Society Open Science. This topic is addressed in more detail in the next section.

Table 1 summarizes some common reasons for excluding studies from systematic reviews and risk assessment. The “Required Information” column reflects critical experimental details that, when not available in papers, may remove them from systematic review rather than just lowering the reliability rating. The “Useful Information” column reflects additional information that can improve a study’s reliability, reduce its overall risk of bias, and increase a study’s chances of being considered in regulatory scientific assessments (Stephens et al. 2016; Thayer et al. 2022; Yost et al. 2019). Finally, we note that many of these and related considerations are addressed in detail in a recent OECD Guidance Document on the Generation, Reporting and Use of Research Data for Regulatory Assessments (Organisation for Economic Co-operation and Development 2025).

## Make data available following the findable, accessible, interoperable, reusable (FAIR) principles

In this new Information Age, researchers are generating more data than ever, and such data are increasingly used with tools like artificial intelligence (AI). This wealth of information might be exceptionally useful if it can be accessed, and examples of its use are beginning to be documented in the popular press and in academic journal articles (Denton et al. 2022; Morgan, 2025 (see Supplemental References); Mumau et al. 2025). This section will briefly illustrate the value of being able to find published data, and then describe how the use of the findable, accessible, interoperable, reusable (FAIR) principles by investigators might support data availability in future research.

An example of the utility of data availability is provided by the discovery of a treatment for a young patient diagnosed with cyclic vomiting syndrome, a disorder that results in frequent episodes of severe nausea and vomiting. The patient’s clinicians exhausted all known treatment options with no symptom relief for the patient. Using an AI tool called mediKanren, a group at the Hugh Kaul Precision Medicine Institute was able to quickly search through treatment options and corresponding publications and to identify a potential treatment that the patient had yet to try: nasal inhalation of isopropyl alcohol. This treatment was shared with the patient’s physician and proved to be successful in alleviating nausea symptoms (Foksinska et al. 2022). While this example is based upon a powerful search of published literature rather than a search of the underlying data itself, it highlights the potential of making knowledge as universally accessible as possible, as well as the value of publishing even seemingly minor research findings. Panelists Rebecca Boyles and Kira Bradford described this exemplary use of AI, but also noted that unfortunately, throughout the research data lifecycle, a variety of challenges often hinder these data from being used to inform decision-making. Much of the data generated are redundant or unintelligible, and are unavailable to key partners (Brett 2022).

Thus, as one navigates this cultural shift toward data sharing and open science, data needs to be collected, managed, curated, and stored in a way that not only ensures data quality and integrity, but also facilitates collaboration, accelerates research and informs health-protective policy. To support use in decision-making and beyond, data needs to be collected and made available following the FAIR principles. The FAIR principles were developed in 2016 to ensure that data are collected and shared in a way that enables and enhances reuse of data by both humans and machines (permitting AI methodology) (Wilkinson et al. 2016). As more funding agencies and publishers begin to require researchers to share data, the FAIR principles have become a vital component of data management and sharing practices.

Making data FAIR and making data open are two closely related, but distinct concepts. Some data simply cannot be shared openly due to restrictions based on ethical, legal or contractual constraints (Jacobsen et al. 2020). As stated in the H2020 Program: Guidelines on FAIR Data, data needs to be “as open as possible and as closed as necessary” (European Commission 2016). Data need to be “open” in order to foster reusability and reproducibility, but at the same time investigators need to be “closed” to protect the privacy and confidentiality of the subjects where necessary (Landi et al. 2020). One may think of FAIR as the toolkit for data management best practices, while open science is the data sharing movement. Embracing FAIR data sharing practices promotes transparency and accelerates scientific advancements by enabling the validation of results and fostering new discoveries. Adopting open science principles encourages wider participation in research efforts, leading to a more inclusive and impactful scientific community.

In environmental health research, a key challenge is effectively collecting, utilizing, and disseminating diverse data types without excluding key partners. Effective environmental management requires accurate data, but it also requires engagement with partners and managing and sharing data in a way that facilitates that engagement (Brett 2022). The shift toward open science via FAIR makes environmental data more available to partners such as regulatory decision-makers and community members by providing a framework for data management and by sharing processes that address key factors to increase the likelihood that research findings might be used in the process of policy development. For example, for data to be reusable they need to be richly described by metadata, including detailed study protocols and methods, and quality controls measures. Datasets that are structured in standard formats, share vocabularies, ontologies, or metadata standards are much more likely to be effectively used. Increased data availability to partners leads to the addition of new information and diverse perspectives, all of which results in more meaningful and productive partner engagement (Beierle 2002). Thus, making data FAIR is essential for evidence-based decision-making as well as for engaging partners effectively, which ultimately contributes to regulatory legitimacy and better environmental decision-making (Brett 2022).

## Development and use of databases to advance and maximize the impact of environmental health research and policy

Making data FAIR enhances the likelihood that partners may find and reuse those data. Investigators might also consider how to best use these data in their research. With the emergence of advanced analytical tools, including machine learning approaches, the utility and importance of comprehensive chemical, toxicological, and exposome databases in generating inferences and hypotheses in environmental health research is rising. Growth of these databases may improve statistical power and validity of inferences for decision-making. Researchers might take advantage of these databases when developing research questions, because these databases may assist in recognizing research gaps and developing hypotheses. Investigators may also design their research and report their data to meet the inclusion criteria for a database(s) of interest. Indeed, the new NIH data sharing policy (National Institutes of Health, n.d.-b (see Supplemental References)) requires researchers to write a Data Management and Sharing Plan precisely such that investigators might consider any submission requirements the repository of interest may have, and thus plan to meet those requirements as part of the study design. While no single protocol exists for a dataset to be included or acquired into a database, a more intentional experimental approach and manuscript presentation may elevate the likelihood of inclusion of a given study into a database. It is noteworthy that some data repositories accept dataset submissions from individual researchers, whereas other repositories identify datasets for inclusion in their collections from peer-reviewed papers or via other means. To promote academic investigators’ use of such databases, their ability to contribute to them in those cases where direct submission is possible, and future academic database development, a number of valuable databases are presented from North America (Table 2), Europe (Table 3), and Asia (Table 4). While not an exhaustive list, these databases provide critical tools for environmental health research, supporting cross-regional comparisons of human and ecological risk thresholds and facilitating mechanistic insights tailored to regionally significant health priorities.

Several other useful databases that are directly relevant to environmental health research, such as the Exposome Explorer database, the ATSDR ToxProfiles, and the Toxic Exposome Database (T3DB), are summarized in Supplemental Table S1.

While every database has advantages, some drawbacks to their use include limitations and challenges in data curation and entry, and lack of integration across multiple platforms and across biological scales. For example, NHANES data provide valuable insights into population level health impacts of chemical exposures but gaining more comprehensive mechanistic or toxicological understanding of specific chemicals often requires integrating additional resources, such as ToxCast and Tox21. However, only a fraction of the NHANES data are integrated with toxicity data, which are accessible through CompTox. Integrating NHANES biomonitoring data with mechanistic and toxicity information from databases like ToxCast and Tox21 might strengthen our ability to predict disease outcomes from multiple environmental exposures, particularly by identifying shared mechanisms or cellular targets. Another challenge with databases, including in database integration, is lack of concordance in data repository requirements and guidelines across databases for emerging peer-reviewed studies. Addressing this remains a critical need for both ensuring rigor in inferences derived through *in silico* data mining efforts and transparency.

Insufficient quality and methodological detail, particularly on experimental parameters, in peer-reviewed articles directly contribute to the need for highly curated and labor-intensive efforts when depositing data. In many cases, the considerations discussed in Table 1 and the section on “Incorporating policy and regulatory needs into experimental design and results reporting” apply here. For example, many databases require raw data from a peer reviewed study to be publicly accessible for it to be considered. For a study to be included in the CTD database, the study needs to be in a peer-reviewed article and have clearly defined and reproducible methods that led to findings relevant to chemical-gene, chemical-disease, or gene-disease interactions. To be considered for the ECOTOX database, a given study needs to include appropriate controls that are explicitly defined to ensure that observed effects are only due to the chemical exposure rather than other variables and must include multiple replicates or independent experiments. Studies also need to adhere to certain testing guidelines (e.g., the US EPA’s or Organization for Economic Co-operation and Development Testing Guidelines) when possible. In contrast, the ToxCast database is not entirely based upon peer-reviewed study data (although methods are peer-reviewed, and many results are peer-reviewed prior to public release) and relies on standardized high-throughput screening data that are directly intended for regulatory toxicology.

In summary, as researchers in environmental health, identifying relevant databases prior to the study might inform hypotheses and experimental design, and in some cases test hypotheses. Adhering to requirements set by databases for data acquisition (at least when possible) might significantly improve the utility of published data for database inclusion. Investigators might also set up their own databases, but it will be critical to do so in a way that these databases are readily shared, are interoperable, and are sustained in the long term. Finally, it is noteworthy that database development and use may be a particularly fruitful area for collaboration between academicians and researchers, with each group bringing different skills and contextual understanding to the collaboration. Recent examples illustrating the practical use of databases where data was leveraged to address regulatory needs include the derivation of 1001 new calibrated toxicity values (estimates of a daily human oral dose likely to be without appreciable risk of adverse non-cancer health effects over a lifetime) (Harrill et al. 2026) and development of a prioritization workflow to identify data-based identification of risk assessment priorities (Environment and Climate Change Canada, 2025b (see Supplemental References)).

## Beyond research: opportunities for direct engagement with regulatory processes

While assuring environmental and toxicological research is usable and accessible for decision-making is a powerful way to engage in the policy process, scientists have additional opportunities to engage more directly in these processes. Submission of public comment letters in response to proposed chemical rulemakings, testifying as an expert witness for public hearings, serving on scientific advisory boards, and directly engaging with regulators or elected officials through in-person meetings are a few ways in which scientists might meaningfully engage in the policymaking process. Regulators indicate that these methods of engagement, whether providing data, making technical recommendations, and/or building a strong scientific case through testimony or shared experience, are particularly effective ways to leverage scientific information in the regulatory process (Topazian et al. 2024).

Submitting public comments is a particularly effective way for scientists to ensure that chemical policy is grounded in the best available science. Through public comments, researchers may highlight key exposure, hazard, and/or risk information that federal agencies need to consider in a proposed regulatory decision, especially information that was missed or will increase protections for public health. According to the Administrative Procedures Act, federal agencies are legally required to respond to every unique, fact-based comment; comment responses provided by regulatory agencies are typically recorded in a “response to comments” document that is made publicly available on the federal register. Even if the agency does not ultimately incorporate data or input from a particular comment, regulators are required to provide justification for their decision in their response to comments. Once a public comment is submitted in response to a particular rulemaking, it remains part of the administrative record and might serve as evidentiary basis for future litigation if that rulemaking were to be challenged in court. For effective comment writing, investigators have recommended that scientists focus on substance over volume, providing evidence that regulators value, helping regulators access hard-to-find information, and speaking to your research expertise (Topazian et al. 2024).

Regulations.gov also advises that concise claims supported by sound reasoning and scientific evidence are best for effective comment writing (Regulations.gov n.d. (see Supplemental References)), and also offers both examples and guidance on how to find opportunities to comment. Through this process, scientists may also glean helpful information regarding what types of data, information, or processes an agency is prioritizing in its work.

Environmental health researchers might submit public comments in response to various scientific and toxicological assessments that are used in decision-making in the US, such as chemical risk evaluations conducted by EPA under the Toxic Substances Control Act, the Agency for Toxic Substances and Disease Registry’s (ATSDR’s) Toxicological Profiles, National Academies of Sciences, Engineering, and Medicine (NASEM) reviews of state-level or other federal exposure, hazard, and risk assessments, and other assessments from EPA programs, such as the Integrated Risk Information System (IRIS) assessments and EPA Risk and Technology Reviews for the National Emissions Standards for Hazardous Air Pollutants (NESHAPs) – among many others.

Other scientific documents that agencies solicit public comment on, such as scientific methodologies or technical guidance documents, are also opportunities for scientists to have direct input on the scientific processes that influence decision-making. For example, EPA published its “Draft Proposed Approach for Cumulative Risk Assessment of High-Priority Phthalates and a Manufacturer-Requested Phthalate under the Toxic Substances Control Act” for public comment in 2023 (US EPA 2023 (see Supplemental References)). This document outlined EPA’s approach to conduct a cumulative risk assessment for phthalates that were undergoing the TSCA risk evaluation process. Researchers from institutions across the US that are part of the Science Action Network (SAN) hosted by the University of California San Francisco’s Program on Reproductive Health and the Environment submitted comments highlighting key scientific issues with the proposed method, and arguing that it failed to consistently reflect the best available science, as required by TSCA. Scientists agreed that dose addition and relative potency factors (RPFs) were appropriate for assessing combined anti-androgenic effects in a cumulative risk assessment, but emphasized the need for more robust systematic review methods and dose-response modeling, more comprehensive evaluation of aggregate phthalate exposures, including combined occupational, consumer, and environmental exposures, and the need to consider additional phthalates, other anti-androgenic chemicals, and non-chemical stressors (e.g., social or environmental factors) that contribute to cumulative risk. In its Draft Phthalate Cumulative Risk Analysis published in December 2024, EPA integrated some of the points highlighted by the SAN (US Environmental Protection Agency 2023, (see Supplementary Reference)).

The scoping phase for TSCA chemical risk evaluations is a critical step in the risk evaluation process when scientific input through public comments might also be highly effective. In this process, the EPA will release a proposed scope document outlining what the risk evaluation will cover, and what hazard and exposure data may be used to support the risk evaluation. Because the scoping phase precedes risk evaluation, scientific input from the public at this stage is critical to catch flaws in proposed methods and point EPA to data that may have been missed, so the risk evaluation is comprehensive and reflective of current evidence.

Similar opportunities exist in other countries. For example, the European Food Safety Authority routinely posts Public Consultations to review draft assessment reports. Respondents are allowed to indicate when relevant studies have not been included.

Participating in federally mandated scientific advisory boards is one of the most direct ways for scientists to give input on agency scientific assessments. For example, EPA has multiple congressionally mandated scientific advisory boards, like Science Advisory Board (SAB), Board of Scientific Counselors, Human Studies Review Board, TSCA’s Science Advisory Committee on Chemicals (SACC), Office of Pesticide Program’s Science Advisory Panel (SAP), and Clean Air Scientific Advisory Committee (CASAC), which provide peer review for scientific assessments used for decision-making at the Agency. Scientific expertise ensures that assessments are grounded in the best available science. Typically, experts are nominated to these committees through a public nomination process, and experts can also self-nominate.

Researchers might also provide expert testimony in other settings. Public hearings organized by elected officials from the local to federal levels may be used to discuss topics relevant to regulatory decisions, including chemical regulations or drinking water standards where scientific expertise is needed and highly valued. Scientists can similarly provide expert testimony to nonprofits. For example, Earthjustice solicited expert testimony from Dr. Russ Hauser and Dr. Ami Zota, both renowned epidemiologists and phthalates experts, to support a litigation to compel FDA to ban phthalates from food packaging. However, it should be noted that serving as an expert witness – particularly if one is paid to do so – might generate a perception of conflict of interest or bias, which scientists should consider.

Further, investigators might meet informally with Agency or Congressional staff to educate decision-makers on a topic that may be relevant to decision-making. For example, researchers from the NIEHS Environmental Health Sciences Core Centers were part of a Hill Day initiative in 2024 to highlight the importance of environmental health research to lawmakers. As part of this initiative, researchers from 25 centers across the US shared research advancing public health and preventing chronic disease. Similarly, researchers might directly engage with regulators and risk assessors and solicit meetings to communicate methods or data that may strengthen regulatory scientific assessments. For example, toxicology experts can solicit meetings with EPA risk assessors from the Office of Chemical Safety and Pollution Prevention (OSCPP) to communicate methods for risk assessment that the Agency may not be relying on but might improve public health outcomes. These conversations enable a line of communication that might align scientific inquiry with real-world regulatory needs and help identify uncertainties or data gaps that are most significant or policy-relevant, allowing researchers to strategically focus their efforts to design studies that are targeted and impactful.

Several established networks provide infrastructure for scientists to engage directly with regulatory science and policy processes. The University of California San Francisco Program on Reproductive Health and the Environment’s (UCSF PRHE) Science Action Network (SAN), a group of over 250 academic scientists, health professionals, and public health experts, provides researchers with the tools, networks, and frameworks investigators need to effectively engage in the regulatory decision-making process. SAN members contribute scientific expertise through public comments, service on federal scientific advisory committees, and targeted input to inform UCSF PRHE’s policy initiatives, including white papers, Congressional briefings, and meetings with Agency staff. The Union of Concerned Scientists maintains a large network of over 20,000 scientists, engineers, and public health professionals who mobilize scientific evidence to strengthen policies that improve health and the environment.

Finally, while it is beyond the scope of this manuscript to address ways for academicians to interact with policymakers and regulators in all countries, such opportunities often do exist in many countries. For example, opportunities also exist in Canada under CEPA for public consultation (Health Canada, 2014 (see Supplemental References)). Examples include 60-day comment periods when a draft screening assessment or Science Approach Document is published including opportunities to submit scientific evidence; comments may be submitted on proposed risk management measures; information gathering notices (Section 71); and nominations of chemicals for assessment (Section 76).

Through proactive engagement with the policy and decision-making processes, scientists might enhance relevance and utility of scientific research in regulatory decision-making and gain insight into what research gaps are most urgent.

## Beyond research: opportunities for direct bi-directional communication with communities

Scientists might interact with community members and/or non-governmental organizations (NGOs) in several ways to bridge gaps between research, policy, and community to advance environmental health.

Individual community members might help researchers understand the community being served, gain exposure to important perspectives, make research more applicable, ensure community needs are prioritized, and drive new research directions (Ramirez et al. 2023). NGOs possess the ability to help investigators align their efforts with local needs and politics, communicate findings in policy settings, and facilitate community engagement due to their proximity to communities and their role as community advocates (Ishaku et al. 2021; Zachariah et al. 2010). Researchers possess the ability to help communities define and understand environmental problems, and put their knowledge to action by providing subject matter expertise to environmental advocates, supporting outreach programs, translating research findings, serving on external advisory boards, testifying for regulatory bodies, coordinating with government scientists, engaging in dialogue with communities, facilitating citizen science, and collaborating on participatory research projects (Cox et al. 2019). While the benefits of engaging with communities are clear, and some excellent guidance on process exists (Simmons 2017), the guidelines for how to engage with community partners are often unclear and may present a challenge to academic researchers (Taffere et al. 2024). The following perspectives, inspired by panelists Alexis Luckey, Grady McCallie, and Frannie Nilsen, representing community groups, NGOs, and government scientists, offer guidance on how academics can effectively communicate with communities to improve environmental health.

When collaborating with community organizations, recognizing what each group brings to the table is important for scientists to interact effectively. For example, advocacy groups often have a wealth of experiences, ranging from community to professional-level involvement. Acknowledging, respecting, and using community expertise is critical to forming relationships and gaining on-the-ground insight from community members. However, mere recognition and acknowledgment are not sufficient to foster authentic engagement, but rather fall under the category of “empty ritual of participation” (Arnstein 1969). Meaningful engagement requires a distribution of power where citizens have significant influence over decision-making processes (Arnstein 1969). In the case of academic-community partnerships, investigator are traditionally the powerholders due to their institutional privilege, funding, and technical expertise. Therefore, researchers are responsible not only for thoughtfully considering how these individuals might benefit and empower a community with their work, but also how these investigators might learn from community members’ expertise by ascribing to an asset-based model of community partnership, which empowers community members to use their skills, lived experiences (including direct exposure), and knowledge to drive change (Kretzmann and McKnight 1993). Forming true partnerships with groups across a wide spectrum ensures that many backgrounds inform the research and collaboration. This range of experience enables researchers to consider which groups complement their needs and goals as scientists. Such thoughtful community partnerships are mutually beneficial and are highly correlated with successful outcomes (Davis and Ramírez-Andreotta 2021).

Scientists should ensure their research is useful to partners through direct, upfront communication. Ensuring that research is useful to the community writ large starts by asking partners such as community members and NGOs how research can be tailored to meet their needs and acting on that input as possible. Authentic engagement requires building long-term relationships, but community collaboration might begin small with simple questions. What environmental health questions are communities interested in having answered? What are their main concerns in their community? How might they like to be involved in design, data collection/analysis, writeup, or dissemination of scientific findings? What resources, recognition, training, and/or compensation might they need to make their contributions possible and worthwhile? After defining a shared research question or goal, researchers and community members need to decide together what community involvement looks like throughout the process; levels of subsequent community involvement might vary widely. Scientists also need to transparently communicate their own limitations, including the relatively slow pace of data generation and analysis, which may surprise community members.

Studies in which research questions were informed by local knowledge are highly associated with tangible changes impacting determinants of health in the community, and thus represent a collaborative and effective way to design impactful research (Davis and Ramírez-Andreotta 2021; Horne et al. 2023). In the words of community members who are part of community-academic partnerships: “Impact is about relationships.” For example, in 2022, the DUSRC CEC formed a relationship with the Waccamaw Siouan STEM Studio, an indigenous community-based and STEM program for tribal youth (Waccamaw Siouan Tribe, n.d. (see Supplemental References)). Out of this relationship came several research questions based upon environmental health concerns within the community. Partners were concerned about PFAS contamination in backyard soils and drinking water. Working collaboratively to address these concerns, the CEC provided connections to researchers, co-created plans for data collection and analysis, helped to create educational resources and materials, and provided an impactful and accessible report-back. A number of DUSRC PIs, trainees, and staff were involved in sample collection, and the Analytical Chemistry Core is analyzing samples.

Establishing such relationships requires a significant time investment to build trust (Cole et al. 2013; Lucero and Wallerstein 2013; Sprague Martinez et al. 2020). In fact, the phrase “moving at the speed of trust,” originally coined by Steven Covey (2006) is now a guidepost to foster meaningful community engagement. Historically, a challenging aspect of this work was that community engagement is not well incentivized or rewarded in academic institutions, though attitudes toward community engagement have shifted positively in the past several decades, leading to increasing institutional support (Klaw et al. 2023; Lucero and Wallerstein 2013, 2013; O’Fallon and Dearry 2002; Trottier et al. 2019; Wilkins and Alberti 2019). Thus, by investing the time and designing studies not only with communities in mind, but in partnership, scientists might generate data that are more likely to be valued by the community and result in public health protection for the community.

To tailor their research outputs for communication with community groups, scientists may focus on producing accessible research outputs that are understandable to a broad audience. Researchers who interpret their findings and disseminate research via publications and conference presentations also need to report back their results to the community that participated in the study, particularly those who provided their own environmental or biological samples (Horne et al. 2023). This needs to be done before sharing results widely to a scientific or a general audience. Respecting community members’ right to hear the results first helps to build trust and allow community members to check the accuracy and thoroughness of the results and ensure that proper credit is given to them. When individual results are shared with research participants, the template of the report must first be reviewed by an Institutional Review Board (IRB) that assesses risk for people engaged in human subjects’ research. The IRB also needs to approve study design prior to initiating the study, among other reasons, to ensure data confidentiality. Transparent communication to community partners should provide an estimate of the time that this will involve. While sharing individual results, data and results need to be conveyed in a manner that does not add undue harm or stress when the health risks cannot truly be understood. In addition, and especially in exposure science research, data and sample ownership also need to be discussed to conduct equitable research and data collection (Horne et al. 2023; Moore, 2023 (for Moore, see Supplemental References)). Further, community-based participatory research (CBPR) results combined with community-first communication strongly motivates behavioral change and adoption of solutions both individually and community-wide (Emmett and Desai 2010).

For clear messaging to communities, researchers should avoid jargon and be explicit regarding the meaning of their findings by reporting results in plain language (Borowiec 2023). Translating research materials to provide guidance on actions to reduce exposure and increase knowledge within communities is essential to promote evidence-based policy for improving health outcomes (Horne et al. 2023). Literal translation (into other languages, such as Spanish) may be important in some contexts as well. When communicating to individuals outside of the scientific community, avoid the usual phrase: “More research is needed.” While this concept is standard in science, it may be read to imply that knowledge in hand is insufficient for action, whether or not that is true. Alternatively, one may contextualize the existing research by providing recommendations based upon the presented information. Information is often best delivered with personal and relatable explanations (i.e., “here’s what this means to me”).

After engaging with a community to produce research findings, scientists should be mindful of whether that material is accessible to partners outside the academic arena. Often, NGOs or community groups do not have access to research that is in peer-reviewed journals and/or behind a paywall. Beyond establishing direct lines of communication between investigators and partners, publishing open access, now a requirement for all NIH-funded research, may help partners to find and use relevant research. In addition, researchers might enhance the reach of their work by issuing a press release alongside a publication (after peer review is complete), which is associated with increased media attention and might improve probability that important research will reach communities and decision-makers (Fuoco et al. 2023). Ultimately, building relationships and trust with outside groups is foundational to the researcher’s ability to understand and meet the needs of communities (Metcalf et al. 2015).

Effective science communication between researchers, communities, and policymakers has led to numerous success stories (Brown et al. 2012; Emmett and Desai 2010; Korfmacher et al. 2016; O’Fallon and Dearry 2002; Petersen et al. 2006; Senier et al. 2008; Sprague Martinez et al. 2017, 2020; Williams et al. 2016). One local example is the uncovering of PFAS contamination in North Carolina’s Cape Fear River due to nearby fluorochemical production at a site on the river (Geosyntec Consultants of North Carolina, 2018 (see Supplemental References)). The discovery of the issue, as well as subsequent efforts to curb contamination and facilitate remediation, required the expertise and collaboration of academic researchers, government agencies (US Environmental Protection Agency (EPA) and the North Carolina Department of Environmental Quality (NCDEQ)), and community/advocacy groups such as Cape Fear River Watch, the Southern Environmental Law Center, and Clean Cape Fear. Research and advocacy have taken the form of publishing peer-reviewed studies, filing petitions, proposing legislation, providing testimonies, and fighting legal battles with responsible parties (North Carolina Department of Environmental Quality, 2022 (see Supplemental References); Schachtman, 2018 (see Supplemental References); Scruggs, 2019 (see Supplemental References) (Sun et al. 2016). The first scientific documentation reporting PFOA contamination in the Cape Fear River occurred in 2007 (Nakayama et al. 2007). In 2009, PFOA began to be replaced with another kind of PFAS, GenX (Scruggs, 2019 (see Supplemental References)). According to results published in 2015, GenX and twelve novel PFAS compounds were detected in the Cape Fear River in samples from 2012–2013 (Nakayama et al. 2007; Strynar et al. 2015). Later, GenX was found in the city of Wilmington’s drinking water and researchers indicated that existing water treatment processes were not equipped to remove the contaminants (Sun et al. 2016). Wilmington’s StarNews reported in 2017 that the local water supply was contaminated with GenX, which was the first time residents were made widely aware of the issue (Hagerty, 2017 (see Supplemental References); Southern Environmental Law Center, 2022 (see Supplemental References); Wickham and Shriver 2021 (see Supplemental References)). This increased public awareness can be largely attributed to direct communication between academic, community, and government partners, as one of the research article’s authors directly emailed the publication to water treatment plants and government officials, including NCDEQ (Wickham and Shriver 2021). Academic partners, with guidance from a Community Science Advisory Board, then responded to community members’ concerns in 2017 by initiating a study to answer questions about their exposure (Kotlarz et al. 2020). Further examples of academic-community partnership include Clean Cape Fear’s use of published scientific findings in congressional testimonies, as well as the co-organization of a PFAS conference by both academics and community activists to foster their continued dialogue (Ohayon et al. 2023). The Southern Environmental Law Center, on behalf of Cape Fear River Watch, sued Chemours, the manufacturer, and NCDEQ in 2018, leading to a consent order requiring the development of a remediation plan (Atwater, 2023; Clean Cape Fear, N.D. (see Supplemental References); Southern Environmental Law Center, 2022 (see Supplemental References)). The mobilization of several nonprofits (Center for Environmental Health, Cape Fear River Watch, Clean Cape Fear, NC Black Alliance, Democracy Green, and Toxic Free NC) in 2020 to petition for PFAS contamination to be addressed demonstrates the capacity of community groups to advance environmental advocacy when the requisite research findings are available (Coastal Review, 2020 (see Supplemental References)). Many of these environmental groups were preexisting and quickly adopted a role in PFAS advocacy, while the PFAS issue also spurred the formation of several new nonprofit activist groups as a direct response to the uncovering of local contamination in 2017 (Wickham 2020). Years of these combined efforts led to the EPA’s proposal for the first national drinking water standards for PFAS in 2023, which highlights the time required for community-engaged work, particularly when seeking policy change (US Environmental Protection Agency 2023). Inspired by the PFAS narrative, programs such as the NCDEQ’s Applied Research Fellowship foster ongoing relationships between government and academia. Such programs are designed to augment the knowledge base of the agency by engaging with leading subject matter experts from local academic institutions, which in turn informs policy to meet the needs vocalized by community members (North Carolina Department of Environmental Quality, 2022 (see Supplemental References)).

In contrast to the traditional model of academic research, community-led, scientist-supported collaborations provide the opportunity for communities themselves to decide what questions they want to be answered. Public input is supremely important, especially given that community members are often the first to recognize and be impacted by adverse outcomes related to environmental contamination (Cohen et al. 2012; Ramirez-Andreotta et al. 2014). Public input provides a perspective that offers crucial lay knowledge, recognizes the applicability of scientific findings to policy, and prioritizes transparent communication of results with communities (Cohen et al. 2012; Ramirez-Andreotta et al. 2014). Ultimately, the “significant renaissance of community engagement in research” (Ahmed and Palermo 2010) speaks for itself in demonstrating the success of participatory methods and the value of centering research on listening to the community and viewing people as research partners rather than research subjects (Davis and Ramírez-Andreotta 2021; Moore, 2023 (see Supplemental References)).

## Obstacles

A number of obstacles exist to researchers’ doing academic research in a way that is useful to regulators, policymakers, nonprofits, and communities.

An overarching obstacle is communication. Regulators, policymakers and concerned citizens often do not have available or established channels to engage with researchers. Conversely, incentives for academic investigators are based largely upon publication in academic journals, where results might be utilized by other researchers. Such publications therefore may include jargon and complex methodologies that are not easily understood and accessible to non-experts. This might hamper understanding by nonscientific partners, especially community groups and NGOs, to engage with or directly apply/benefit from the research findings (Davies and Nutley 2008; Lomas 2005).

Beyond communication barriers, obstacles exist within each step of the research process. The following paragraphs describe obstacles in idea generation, project funding, data generation, drawing conclusions, and data sharing.

### Idea generation

Academic research is often driven by the goal of expanding basic science knowledge, and thus frequently may have limited short-term utility to a general audience, and/or ability to inform large scale policy decisions (Nanglu et al. 2023). While some scientists aspire for their research to be used in a translational manner, or are versed in community-engaged research practices, answering fundamental research questions regarding how nature works and its underlying laws does and needs to remain a top priority (Cairney and Oliver 2017; Fazey et al. 2013; Head 2022; Marshall 2025; National Academies of Sciences, Engineering and Medicine, 2017 (see Supplemental References)). Thus, the hypotheses that most investigators are studying may may generate data that are not be immediately (in the short term) applicable or translatable in all cases. In addition, research aimed at addressing community or policy needs may not necessarily be hypothesis-driven, may be more exploratory in nature, and may require a more transdisciplinary approach. Academic silos can make it difficult for researchers in different fields (e.g., toxicology, public health, economics) to collaborate effectively. This might further hinder the production of research that is both comprehensive and applicable across multiple sectors. Overall, the research goals and methodologies used might create a gap between academic research and practical needs of regulators or policymakers who are looking for actionable insights or evidence-based solutions. However, as discussed in preceding sections, in some cases, hypothesis-based and mechanistic work might also be highly applicable (Figure 1).

### Project funding

Questions that researchers investigate are heavily influenced by their funding source. Academic research in biomedical fields, including toxicology, is primarily funded through grants by the federal government, particularly agencies like the National Institutes of Health (NIH) and Environmental Protection Agency (EPA). Grants that get funded typically propose hypothesis-driven research that has substantial evidence supporting the hypothesis. Within toxicology and environmental health, a powerful and unusual example of a program that funds research that incorporates both fundamental and applied research is the Superfund Research Program (SRP) (National Institute of Environmental Health Sciences (see Supplemental References), 2024; US Environmental Protection Agency, 2014 (see Supplemental References)). SRP grants are designed to support research related to hazardous waste sites, environmental contaminants, and their effects on public health. Most of the research funded by these grants is hypothesis-driven and typically also focuses on areas where there is a clear scientific need. In addition, all SRP centers are required to include a Community Engagement Core (CEC), which provides funding to support community engagement, a Research Translation Core to make results more accessible, a Data Management and Analysis Core to ensure data rigor and availability, and work to develop solutions for communities to reduce or mitigate the impact of exposure to hazardous substances. A similar NIEHS mechanism funds Environmental Health Sciences Core Centers (EHSCC) that conduct, fund, and translate environmental health research that benefits communities. EHSCCs also require a CEC. However, outside of these funding mechanisms, such requirements are quite uncommon, making it more difficult for researchers to fund more applicable research. In some cases, universities may have research translation, communication, and community engagement offices or resources that can be utilized.

Instances also exist where private interest groups provide funding for research; these present different kinds of obstacles and opportunities. For example, pharmaceutical companies, chemical manufacturers, and agricultural companies have historically funded research to examine the safety of their products. Similarly, environmental advocacy and other nonprofit groups may fund research to further causes like advocating for stricter regulatory standards or to challenge specific public health claims. This sometimes leads to concerns about conflicts of interest or the potential for research bias. A notable example is the funding provided by tobacco companies to scientists studying the health effects of smoking (Barnes and Bero 1998; Hirshbein 2012)). Even disclosed financial conflicts of interest are reported to influence outcomes (Fabbri et al. 2018; Lundh et al., n.d.; Odierna et al. 2013; Psaty and Kronmal 2008; Psaty and Prentice 2010). Mebane et al. (2019) and Walker and Roberts (2018) describe critical scientific integrity issues associated with funding sources and other real or perceived conflicts of interest. While scrutinizing potential conflicts of interest is important, dismissing research from a particular sector out of hand fosters an uncritical skepticism that might hinder scientific progress. To guard against possible bias when accepting private research funding, academic researchers should uphold the highest ethical standards and remain committed to rigorous, unbiased science. The credibility of research – whether conducted in academia or industry – relies on transparency, reproducibility, and integrity in both methods and reporting. By consistently prioritizing sound scientific practices, academic researchers not only strengthen public trust but also provide a critical benchmark that holds all sectors accountable. In doing so, they help create a more informed and balanced dialogue around evidence, ensuring that knowledge advances based on merit rather than assumptions about origin. Upholding these values reinforces the role of academia as a cornerstone of objective inquiry and societal progress, while also increasing the likelihood that such research is widely useful.

Even in cases where funding for more applied or community-engaged work is secured, the grant’s timeline or budget may be insufficient to engage with communities to a level or for the time necessary to support community needs/interests. Federal and private sector grants often come with fixed timelines (typically one to five years) that are often too short for researchers to build sustained, trust-based relationships with the communities they aim to work with and study, especially in areas affected by environmental hazards like Superfund sites. To circumvent this obstacle, sustained support from the academic institutions hosting the research or donor individuals’ organizations is needed to prevent a breakdown in relationships between the researchers and key community partners.

Finally, funding priorities for governmental and non-governmental sources change over time. This can be an obstacle if funding is no longer available, but may also present opportunities. For example, recent increased emphasis by the NIH on rigor, reproducibility, research translation, and open access to both data and published manuscripts are in full alignment with suggestions in this manuscript.

### Data generation

In order for data generated at the bench to be useful outside of the academic sphere it must be both reproducible and generalizable (Mebane et al. 2019). Research results that cannot be replicated or applied to a wide range of contexts are of limited value in informing policy or improving public health. Toxicological experiments in particular should be designed such that the conditions tested are as relevant to current environmental conditions and reflective of real-world exposure scenarios as possible. For instance, laboratory studies typically involve exposure levels above environmental concentrations to ensure a measurable biological response, which is useful for mechanistic inquiry but requires extrapolation to infer lower-dose effects. This creates an apparent disconnect between laboratory findings and human health risks that can complicate risk assessment and requires explanation for non-technical audiences. As previously mentioned, another challenge is finding ways to connect research on one chemical at a time to the reality of exposure to hundreds of chemicals simultaneously (Drakvik et al. 2020; Hernández and Tsatsakis 2017). Last, some of the experimental design considerations addressed earlier, such as the use of a wide range of doses and multiple endpoints, may be challenging to comply with given funding constraints (i.e. not enough funds to test biological effects at multiple doses, and reviewers may not view this as a priority). Ultimately, however, investigators need to carry out research that is of the highest possible quality, which might enhance the likelihood that their research is useful to policy-makers, communities, and others.

### Data analysis and translation

Data analysis and presentation with community partners may be challenging because additional time and skills are needed for the non-technical interpretation and translation of data in a way that contextualizes and communicates the findings for the specific communities they affect (Cashman et al. 2008). Similar constraints apply to communicating and establishing data analysis practices developed in academia with regulators. Regulatory scientists, like academicians, are often engaged in the development and standardization of methods to analyze and interpret complex datasets, but without clear guidance/best practices that researchers and assessors can consistently apply, use will be limited to case-by-case dependent scenarios. Some funding agencies and programs, including the SRP and EHSCC, support research translation efforts, but most do not; thus, funding is an obstacle.

### Drawing conclusions and publishing

A significant obstacle to translating academic research into policy actions lies in the pressures that investigators face within academia, particularly the drive to publish frequently to secure tenure, demonstrate productive use of previous research funding, and obtain future research funding (Rawat and Meena 2014). Community service and engagement is not often highly valued during the academic promotion review. Further, in addition to the previously mentioned tendency to not publish negative data, data that do not reproduce previous results are challenging to publish (DeVito and Goldacre 2019; Dwan et al. 2013; Ioannidis 2005; Mahoney 1977; Rosenthal 1979), and obtaining funding to test reproducibility might also be difficult. This may lead to selective publication of results that correspond with the current literature and hence may alter the broader impact of the research by biasing the overall literature and databases (van Dalen and Henkens 2012). Such biases have a downstream effect on the accuracy of the toxicological knowledge available for regulators, policymakers, nonprofits, and communities to use.

### Data sharing

Again, traditionally, research findings are disseminated through scientific journals or academic conferences, which are often inaccessible to non-scientists due to their technical language and paywall restrictions (Nutley, Walter, and Davies 2007). New NIH policies regarding open access publication partially alleviate the latter concern (National Institutes of Health n.d. (see Supplemental References)). Research findings may be made more accessible with research translation techniques such as infographic brochures or community meetings. However, reporting back research results to impacted communities involves significant logistical, methodological, and financial challenges. As discussed, grants rarely allocate funds for outreach activities to communicate findings to non-expert audiences. Also, regulatory barriers may exist to reporting research results, particularly when health data are involved. In studies where personal health information is collected researchers must navigate complex privacy regulations, such as HIPAA (Health Insurance Portability and Accountability Act) in the United States, which protects the confidentiality of personal health information (Shen et al. 2006). In some cases, IRB programs may limit the sharing of research results relating to chemical exposures or health measures because these originate from a non-CLIA (Clinical Laboratory Improvement Amendments) certified lab. CLIA certification ensures that lab protocols are thoroughly documented and use the highest standards for measurements, ensuring that testing is accurate and reliable. However, non-CLIA certified labs, which are common in academic research, are also capable of producing high-quality data and results but may face financial and resource limitations in achieving this certification. This might limit the researchers’ ability to report findings to study participants or affected communities. While these regulatory requirements are critical for protecting private data and ensuring data quality, these nonetheless also comprise logistical and financial obstacles to data sharing. Compromises need to be reached by which data are shared with study participants with the acknowledgment that the lab is non-CLIA certified and a recommendation that results be shared with their medical provided and, if necessary, re-testing performed. If we don’t share these results, we risk losing the trust of our communities.

## Conclusions

Environmental health research produces both fundamental new understandings of how the world works, with important long-term benefits, and specific data that are critically important in the short term for decisions made by policymakers, decision-makers, communities, and others. Both outcomes are important and valuable in and of themselves. However, it is hoped that this review might provide useful guidance to researchers who seek to identify ways that investigators might do both at once. It is anticipated that these might constitute iterative practices in which research questions and experimental designs that serve both goals are identified, research outcomes are shared and communicated, and needs are revisited. In this way, such research might serve a third function – strengthening relationships between researchers and the larger society.

## Supplementary Material

Supp 2

Supp 1

Supplemental data for this article can be accessed online at https://doi.org/10.1080/10937404.2026.2636513.

## Figures and Tables

**Table 1.** Common exclusion and inclusion criteria found in literature search screens.

	Required Information	Useful Information

Reporting of Experimental Design	Species Sex (specifying sex of test subjects is required by some agencies; inclusion of male and female required in some cases) Age Test method description and citation if applicable Material Source Chemical purity Experimental conditions identical across study groups Inclusion of negative control Number and range of doses/concentrations Duration of exposure Route of Exposure Exposure administration consistent across study groups Statistical analysis methodology	Pre-published study protocol Strain Inclusion of positive control Husbandry Procedures Description of vehicle/negative control Description of cell culture media Environmental conditions (temperature, lighting, oxygen content, etc.)
Bias Mitigation	Random group assignment Blinded allocation to study groups Blinded research personnel during data collection (for qualitative and quantitative outcomes) Declaration of financial conflicts of interest	Process for randomizing subjects/samples into experimental groups Declaration of blinded allocation to study groups Declaration of blinded research personnel during data collection (and explanation if not appropriate, e.g. when data is collected using instrumentation that does not require human intervention)
Confounding Variable Mitigation	Explanation for results with lower number of subjects than described in methods	Record of reasons for subject/sample attrition
Reporting of Results	All results and pre-specified outcomes reported, including negative findings and positive and negative controls	Availability of data underlying graphs

**Table 2.** Toxicology and chemical data resources overview.

Database/Resource	Description	Key Strengths	Limitations/Notes

ToxCast/Tox21	Major repository of *in vitro* toxicological data for ~8,500 chemicals using high-throughput biological assays (Richard et al. 2021).	Broad chemical coverage; integrates with computational models; EPA/NIH/FDA collaboration.	Primarily predictive; *in vitro* focus; flags to indicate quality concerns; cytotoxicity should be considered when interpreting.
ToxRefDB	Database of *in vivo* guideline or guideline-like toxicity studies for over 1100 chemicals.	Detailed study design and dose-response data; supports validation of High Throughout Screening methods.	Limited chemical scope; based on traditional animal testing.
CompTox Chemicals Dashboard	EPA platform integrating physicochemical, environmental fate, and toxicological data on ~ 1 million chemicals.	Strong data integration across ToxCast/ NHANES etc.; advanced search; supports chemical prioritization.	Many data are model-based; incomplete experimental toxicity data.
IRIS	EPA program providing human health hazard assessments and toxicity values.	High regulatory credibility; consistent peer-reviewed assessments.	Limited chemical coverage, emphasis on historical not emerging chemicals; relatively slow update cycles.
Comparative Toxicogenomics Database (CTD)	Curated database of chemical-gene-disease interactions supporting mechanistic toxicology.	Strong pathway and disease association insight potential; expert curation.	Limited exposure data; focus on gene-disease interactions.
ECOTOX	EPA database of ecological toxicity data for aquatic and terrestrial species.	Essential for environmental risk assessment; peer-reviewed curation.	Limited integration with human health databases.
NHANES	CDC data providing population-level biomonitoring and health outcome data.	Critical for exposure assessment and epidemiology.	Primarily US focused; not mechanistic.
HSDB (via PubChem)	Peer-reviewed information on toxicity, safety, and environmental fate of hazardous substances.	High data reliability, scientific peer review; comprehensive profiles.	Lacks high-throughput and predictive toxicology data.
SeqAPASS	EPA tool for predicting cross-species susceptibility using protein sequence similarity.	Supports NAMs; fills ecological data gaps.	Relies on existing data; reliance on sequence similarity alone limits prediction accuracy.
CEPA Domestic Substances List (DSL)	Canadian inventory of substances manufactured or imported into Canada under CEPA.	Broad national chemical coverage; supports regulatory prioritization.	Not toxicity-based; limited hazard information.
Canadian Health Measures Survey (CHMS)	National Canadian biomonitoring survey measuring chemicals in blood and urine.	Representative exposure data; supports public health assessments.	Limited toxicity endpoints; correlation not causation.
EAS-E Suite	Integrated exposure and safety estimation platform used by the Canadian government.	Combines exposure, fate, and hazard screening tools.	Screening-level modeling; not a substitute for experimental data.

**Table 3.** European toxicology and chemical data resources.

Database/Resource	Description	Key Strengths	Limitations/Notes

ECHA CHEM/REACH (Registration, Evaluation, Authorization and Restriction of Chemicals) Dossiers	Regulatory database maintained by the European Chemicals Agency (ECHA) containing hazard, use, and risk information for over 100,000 substances registered under REACH.	Comprehensive regulatory coverage; standardized hazard classifications; strong legal and scientific basis.	Primarily regulatory-focused; variable data completeness; limited high-throughput screening data.
European Chemical Biology Database (ECBD)	Open-access database adhering to FAIR principles, providing high-throughput screening and chemical biology data for thousands of compounds.	Over four million data points; supports mechanistic toxicology, hypothesis testing, and drug discovery.	Primarily research-oriented; limited exposure and population-level context.
IPCHEM (Information Platform for Chemical Monitoring)	European Commission - supported platform integrating chemical monitoring data across environmental, food, human biomonitoring, and indoor sources.	Cross-domain exposure comparisons; visualization tools; centralized EU access point.	Heterogeneous data structures and quality; limited harmonization across sources.
HBM4EU Dashboard	European Human Biomonitoring Initiative platform providing harmonized biomonitoring data for priority substances across Europe.	Highly coordinated study design; strong data harmonization; supports risk assessment and policy.	Limited to selected priority chemicals; not designed for mechanistic inference.
EU ChemPortal	European Union chemical information portal providing access to multiple EU chemical databases and regulatory resources.	Centralized access point; improves discoverability of EU chemical data resources.	Primarily a gateway; limited standalone analytical or toxicological data.

**Table 4.** Asian toxicology and chemical data resources.

Database/Resource	Description	Key Strengths	Limitations/Notes

NITE-CHRIP (Japan)	Chemical Risk Information Platform managed by Japan's National Institute of Technology and Evaluation, providing hazard, risk, and regulatory information similar to ECHA CHEM.	Comprehensive hazard and risk data; includes GHS classifications, physicochemical properties, toxicological and ecotoxicological results.	Primarily regulatory-focused; variable depth across chemicals.
JECDB (Japan Existing Chemical Database)	Searchable database of toxicity test reports for existing chemicals in Japan.	Covers acute, chronic, reproductive, mutagenicity, and other toxicity endpoints.	Limited exposure or biomonitoring context.
Japanese Human Biomonitoring (HBM) Database	Ministry of Environment - supported surveys of human chemical exposure across multiple chemicals.	Provides population-level exposure data; supports trend analysis.	Some data access restrictions; limited linkage to health outcomes.
Health & Chemicals Portal (Japan)	Portal providing access to ecotoxicity and environmental chemical data.	Supports environmental risk assessment and regulatory review.	Primarily ecotoxicity-focused; limited human health integration.
J-CHECK/AJCSD (Japan)	Integrated platforms combining multiple Japanese chemical safety databases for aggregated searches.	Enables cross-database queries and consolidated chemical safety information.	Dependent on source database completeness; limited predictive modeling.
NCIS/K-REACH (South Korea)	National Chemical Information System and K-REACH portals providing consolidated regulatory and hazard data.	Strong regulatory alignment; centralized access to chemical information.	Focus on regulated chemicals; limited research-oriented data.
KoNEHS (Korea National Environmental Health Survey)	National human biomonitoring program providing population-representative chemical exposure data since 2009.	Robust longitudinal exposure data; supports trend and distribution analyses.	Exposure-focused; limited mechanistic toxicology data.
Hazardous Substances Database (Singapore, EPMA)	Public database listing regulated hazardous substances, authorizations, and restrictions.	Clear regulatory guidance; transparent access to controlled substances lists.	Limited toxicological depth; regulatory scope only.
Australian Human Biomonitoring Programme	National program providing aggregate reports on population-level chemical exposure.	Supports public health assessment and exposure trend analysis.	Aggregate reporting limits chemical-specific or mechanistic analysis.
Hong Kong Hazardous Chemicals Control & Toxic Substances Monitoring	Regulatory frameworks and monitoring programs for hazardous chemicals in Hong Kong.	Supports regulatory oversight and environmental monitoring.	Limited centralized database access; fragmented data sources.
Taiwan TCCSCA Database & Chemical Substance Registration System	Taiwan EPA - supported databases managing toxic and concerned chemical substances and registrations.	Strong regulatory coverage; supports chemical tracking and control.	Limited public toxicological detail; regulatory focus.
China IECSC & Catalog of Hazardous Chemicals	National inventories of existing and hazardous chemicals managed by Chinese authorities.	Extensive coverage of chemicals in commerce; foundational regulatory tools.	Limited public access to detailed toxicity or exposure data.

## Data Availability

No primary data are associated with this review.
