# Supplementary material for: How can academic researchers more effectively contribute to environmental toxicology and health efforts for regulatory decisions, policymakers, nonprofits, and communities?
Source: J Toxicol Environ Health B Crit Rev. Author manuscript; Available in PMC 2026 Apr 1. (PMC13036803; doi:10.1080/10937404.2026.2636513)
Supplement: Supp 2 [file NIHMS2157329-supplement-Supp_2.docx]

# **Supplemental Table 1: Additional database resources**

| Name | Web link | Comments |
| --- | --- | --- |
| Blood Exposome database | <https://bloodexposome.org/> | The Blood Exposome Database is a catalogue of the chemicals (endogenous and exogenous) that are expected and detected in human blood specimens. The database was created using a text mining approach using the [NCBI PubChem](https://pubchem.ncbi.nlm.nih.gov/) , [NCBI PubMed](https://pubmed.ncbi.nlm.nih.gov/) and [NCBI PMC](https://www.ncbi.nlm.nih.gov/pmc/) databases. Chemicals that have been reported in the primary literature (original research articles) for blood specimens are included in the database. Additionally, data from biomonitoring surveys and metabolomics datasets (publicly available) for human blood specimens are also covered in the database. |
| Chemical Entities of Biological Interest (ChEBI) | <https://www.ebi.ac.uk/chebi/> | ChEBI is a dictionary of molecular entities. It focuses primarily on small chemical compounds that intervene in the biological processes of living organisms. Currently, it has over 60 000 annotated compounds |
| Exposome Explorer database | <http://exposome-explorer.iarc.fr/> | The Exposome Explorer database is dedicated to biomarkers of exposure to environmental risk factors for diseases. |
| NASA | [https://genelab.nasa.gov/](https://urldefense.com/v3/__https:/genelab.nasa.gov/__;!!OToaGQ!onty8nokZv3Ui_a_SZcyNOC6kZK5jw72axFCUCc6_xSCWgi2dP1XOP3XCLf4QcZSIPaEMXF92zzRG66BqWDUnAj_EWaaWArKe1k$) | This is a niche database of data from space research. It particularly has a lot of data on radiation exposure. |
| National Center for Biotechnology Information (NCBI) | <https://www.ncbi.nlm.nih.gov/gds/> | NCBI's Gene Expression Omnibus (GEO) primarily focuses on gene expression data, but it does host datasets related to environmental health, particularly when gene expression studies intersect with environmental exposures. This was mentioned by every member of the panel during the workshop. |
| National Center for Biotechnology Information (NCBI) | <https://www.ncbi.nlm.nih.gov/pmc/articles/PMC10857773/> | Table 1 in this article lists many more databases. |
| Pharos | <https://pharos.habitablefuture.org/> | Pharos provides hazard, use, and exposure information on more than 200,000 chemicals used in the materials economy. |
| Recount3 database | <https://rna.recount.bio/> | The recount2 resource is composed of over 70,000 uniformly processed human RNA-seq samples spanning TCGA and SRA, including GTEx. While Recount3 itself does not focus on environmental data, it may contain gene expression data from studies involving environmental health, depending on the original context of the RNA-seq experiments. |
| Toxic Exposome database (T3DB) | <http://www.t3db.ca/> | The Toxic Exposome Database (T3DB) - The database currently houses 3678 toxins described by 41 602 synonyms, including pollutants, pesticides, drugs, and food toxins, which are linked to 2,073 corresponding toxin target records. Altogether there are 42 374 toxin, toxin target associations. Each toxin record (ToxCard) contains over 90 data fields and holds information such as chemical properties and descriptors, toxicity values, molecular and cellular interactions, and medical information. |
