# Supplementary material for: How can academic researchers more effectively contribute to environmental toxicology and health efforts for regulatory decisions, policymakers, nonprofits, and communities?
Source: J Toxicol Environ Health B Crit Rev. Author manuscript; Available in PMC 2026 Apr 1. (PMC13036803; doi:10.1080/10937404.2026.2636513)
Supplement: Supp 1 [file NIHMS2157329-supplement-Supp_1.docx]

# Supplemental references

Meyer et al., “How can academic researchers more effectively contribute to environmental toxicology and health efforts for regulatory decisions, policymakers, nonprofits, and communities?” Journal of Toxicology and Environmental Health, Part B: Critical Reviews.

Note: these references are from non-peer-reviewed sources, which is why they are listed separately in supplemental materials.

Abraha, R. (2019, October 22). The 21-Year Gap. *Understanding Houston*. <https://www.understandinghouston.org/blog/the-21-year-gap>

ARC Arnot Research and Consulting. (n.d.). Eas-E-Suite. *ARC Arnot Research & Consulting*. Retrieved January 7, 2026, from <https://arnotresearch.com/eas-e-suite/>

Atwater, W. (2023, November 7). Forever chemicals, forever concerns: Cape Fear River’s ongoing PFAS problem. *North Carolina Health News*. <https://www.northcarolinahealthnews.org/2023/11/07/forever-chemicals-forever-concerns-cape-fear-rivers-ongoing-pfas-problem/>

Clean Cape Fear. (N.D.). *Timeline of PFAS in the Cape Fear River*. <https://www.cleancapefear.org/timeline-1>

Coastal Review Staff. (2020, October 14). NC Nonprofits Petition EPA over PFAS. *Coastal Review*. <https://coastalreview.org/2020/10/nc-nonprofits-petition-epa-over-pfas/>

ECHA. (n.d.). *Information requirements: 1 to 10 tonnes per year - ECHA*. Retrieved January 7, 2026, from <https://echa.europa.eu/support/registration/what-information-you-need/information-requirements-10-tn>

Environment and Climate Change Canada. (2025, November 4). *Science approach document - Chemical screening and prioritization: Health Canada’s automated workflow for prioritization (HAWPr)* [Assessments]. <https://www.canada.ca/en/environment-climate-change/services/evaluating-existing-substances/science-approach-document-health-canada-automated-workflow-prioritization.html>

Environmental Defense Fund. (2025). *U.S. Climate Vulnerability Index*. The U.S. Climate Vulnerability Index. <https://climatevulnerabilityindex.org/>

Geosyntec Consultants of North Carolina. (2018, September 17). *Assessment of the Chemical and Spatial Distribution of PFAS in the Cape Fear River*. <https://www.chemours.com/en/-/media/files/corporate/cape-fear-river-pfas-report-2018-09-17.pdf?rev=884aaff580a948998a351b45cde646ea&hash=3B774DFCCC8110B408D587D52F65D3A5>

Hagerty, V. (2017, June 7). *Toxin taints CFPUA drinking water*. <https://www.starnewsonline.com/story/news/environment/2017/06/07/toxin-taints-cfpua-drinking-water/20684831007/>

Health Canada. (2014, December 3). *Identification and selection of priorities for assessment under CEPA* [Program descriptions]. <https://www.canada.ca/en/health-canada/services/chemical-substances/chemicals-management-plan/initiatives/identification-chemicals-polymers-risk-assessment-priorities.html>

Moore, E. B. (2023, April 6). Healthy Soils, Healthy Communities: Utilizing Culturally-Responsive Soil Science to Promote Environmental Health in Marginalized Communities – Integrated Toxicology & Environmental Health Program. *Integrated Toxicology & Environmental Health Program*. <https://sites.nicholas.duke.edu/envhealth/seminars-symposia/spring2023seminar/06apr2023/>

Morgan, K. (2025, March 20). Doctors Told Him He Was Going to Die. Then A.I. Saved His Life. *The New York Times*. <https://www.nytimes.com/2025/03/20/well/ai-drug-repurposing.html>

National Academies of Sciences, Engineering and Medicine. (2017, April). *Revitalizing the University-Industry-Government Partnership: Creating New Opportunities for the 21st Century: Proceedings of a Workshop–in Brief" at NAP.edu*. <https://doi.org/10.17226/25080>

National Institute of Environmental Health Sciences. (2024, November 26). *Superfund Research Program*. National Institute of Environmental Health Sciences. <https://www.niehs.nih.gov/research/supported/centers/srp>

National Institutes of Health. (n.d.-a). *Data Management and Sharing Policy | Data Sharing*. Scientific Data Sharing. Retrieved March 31, 2025, from <https://sharing.nih.gov/data-management-and-sharing-policy>

National Institutes of Health. (n.d.-b). *NIH Public Access Policy Overview*. Public Access Policy Details. Retrieved January 7, 2026, from <https://nihodoercomm.az1.qualtrics.com/jfe/form/SV_eypqaXlx2j1IY9T?Q_CHL=si&Q_CanScreenCapture=1>

NC Department of Health and Human Services • Division of Public Health. (2026, January 5). *North Carolina Fish Consumption Advisories*. ArcGIS StoryMaps. <https://storymaps.arcgis.com/stories/eab23dd84e8b403b95dc1223703aa487>

North Carolina Department of Environmental Quality. (2022a). *RELEASE: DEQ and NC Collaboratory Announce Fellowship Program*. <https://www.deq.nc.gov/news/press-releases/2022/06/17/release-deq-and-nc-collaboratory-announce-fellowship-program>

North Carolina Department of Environmental Quality. (2022b). *Settlement ends Chemours challenge of permit to reduce PFAS entering the Cape Fear River*. <https://www.deq.nc.gov/news/press-releases/2022/11/14/settlement-ends-chemours-challenge-permit-reduce-pfas-entering-cape-fear-river>

OECD. (n.d.). *eChemPortal: Global Portal to Information on Chemical Substances*. OECD. Retrieved January 7, 2026, from <https://www.oecd.org/en/data/tools/echemportal-global-portal-to-information-on-chemical-substances.html>

Owens, R. (2019, April 19). UTSW scientists map life expectancy of Texans by area, race, and gender—CT Plus—UT Southwestern. *CenterTimes Plus*. <https://www.utsouthwestern.edu/ctplus/stories/2019/life-expectancy-texas.html>

Regulations.gov. (n.d.). *Tips for Submitting Effective Comments*. <https://s3.amazonaws.com/prod-regulations-faq/pdf/Tips-For-Submitting-Effective-Comments.pdf>

Schachtman, B. (2018, October 29). Scientist says GenX legislation based on his work is good start, but limited by lobbyists. *Port City Daily*. <https://portcitydaily.com/local-news/2018/10/29/scientist-says-genx-legislation-based-on-his-work-is-good-start-but-limited-by-lobbyists/>

Scruggs, S. (n.d.). PFAS — a problem in North Carolina drinking water. *Fluoride Action Network*. Retrieved January 7, 2026, from <https://fluoridealert.org/news/pfas-a-problem-in-north-carolina-drinking-water/>

Shapiro-Garza, E. (2025, July 14). *CEC Works with Cape Fear River Basin Partners to Revise “Stop, Check, Enjoy” – Duke University Superfund Research Center*. Duke Superfund Community Engagement Core. <https://sites.nicholas.duke.edu/superfund/community-engagement-core-cec-works-with-cape-fear-river-basin-partners-to-revise-the-stop-check-enjoy-campaign/>

Shapiro-Garza, E., Rajaee, M., Cohen, S., Klein, C., & Joyce, A. (2022, June). *Subsistence Fish Consumption on the Lower Cape Fear River: Summary of Research 2016 – 2022*. Duke University Superfund Research Center. <https://sites.nicholas.duke.edu/superfundcec/files/2022/10/Subsistence-Fish-Consumption-on-the-lower-Cape-Fear-River_report.pdf>

Southern Environmental Law Center. (2022). *North Carolina GenX and other PFAS Timeline*. SouthernEnviroment.org. <https://www.southernenvironment.org/wp-content/uploads/2022/12/2022-12-05-SELC-timeline-NC-GenX-PFAS-pollution-Chemours.pdf>

University of California, San Francisco program on Reproductive Health and the Environment. (2023, 27). *Comment submitted by University of California, San Francisco Program on Reproductive Health and the Environment (UCSF PRHE)*. Cumulative Risk Assessment; Science Advisory Committee on Chemicals (SACC) Virtual Public Meeting; Notice of Availability and Request for Comment. <https://www.regulations.gov/comment/EPA-HQ-OPPT-2022-0918-0040>

US Court of Appeals. (2024, August 27). *Corrosion Proof Fittings v. EPA, 947 F.2d 1201 (5th Cir. 1991)*. Justia Law. <https://law.justia.com/cases/federal/appellate-courts/F2/947/1201/153685/>

US Environmental Protection Agency. (2014, July 11). *Superfund* [Collections and Lists]. <https://www.epa.gov/superfund>

US Environmental Protection Agency. (2024, April 26). *PFAS National Primary Drinking Water Regulation*. Federal Register. <https://www.federalregister.gov/documents/2024/04/26/2024-07773/pfas-national-primary-drinking-water-regulation>

US Environmental Protection Agency, O. (2021, December 6). *Consideration of Chemical Co-exposure in TSCA Risk Evaluations* [Overviews and Factsheets]. Scientific Advisory Board. <https://archive.epa.gov>

Waccamaw Siouan Tribe. (n.d.). *Waccamaw Siouan STEM Studio*. Retrieved September 9, 2025, from <https://stemstudio.waccamaw-siouan.org/>
